# Operability, Acceptability, and Usefulness of a Mobile App to Track Routine Immunization Performance in Rural Pakistan: Interview Study Among Vaccinators and Key Informants

**DOI:** 10.2196/16081

**Published:** 2020-02-13

**Authors:** Shehla Zaidi, Saqib Ali Shaikh, Saleem Sayani, Abdul Momin Kazi, Adeel Khoja, Syed Shahzad Hussain, Rabia Najmi

**Affiliations:** 1 Division of Woman and Child Health Aga Khan University Karachi Pakistan; 2 Department of Community Health Sciences Karachi Pakistan; 3 Department of Health Government of Sindh Karachi Pakistan; 4 E-Health Resource Centre Aga Khan Development Network Karachi Pakistan; 5 Department of Medicine Aga Khan University Karachi Pakistan; 6 Department of Community Health Sciences Aga Khan University Karachi Pakistan

**Keywords:** mHealth, immunization, digital technology, experience, health workers

## Abstract

**Background:**

There has been a recent spate of mobile health (mHealth) app use for immunizations and other public health concerns in low- and middle-income countries. However, recent evidence has largely focused on app development or before-and-after effects on awareness or service coverage. There is little evidence on the factors that facilitate adoption of mHealth programs, which is critical to effectively embed digital technology into mainstream health systems.

**Objective:**

This study aimed to provide the qualitative experiences of frontline health staff and district managers while engaging with real-time digital technology to improve the coverage of routine childhood immunization in an underserved rural district in Pakistan.

**Methods:**

An Android-based app was iteratively developed and used for a 2-year period in 11 union councils of the Tando Muhammad Khan district, an underserved rural district with poor immunization coverage in Pakistan. We used iterative methods to examine the (1) acceptability and operability of the app, (2) validity of the collected data, and (3) use of the collected data. In addition, we collected the barriers and enablers for uptake of the mHealth app. Each of these topics was further explored related to changes in work as well as the enabling factors for and barriers to app use. In-depth interviews were conducted with the 26 vaccinators posted in the 11 union councils and 7 purposively selected key informants (government district managers) involved with the Expanded Program for Immunization. Findings were triangulated in line with the three broad research areas.

**Results:**

Digital immunization tracking was considered acceptable by vaccinators and district managers. Real-time immunization data were used to monitor vaccination volume, track children with incomplete vaccinations, develop outreach visit plans, correct existing microplans, and disburse a fuel allowance for outreach sessions. The validity of the app data was perceived to be superior to that of data from manual records. Ease of operability, satisfaction with data, personal recognition, links to field support, and a sense of empowerment served as powerful enablers. Taking twice the time to complete both manual and digital entries and outdated phones over time were considered constraints. An unintended knock-on effect was improved coordination and strengthening of Expanded Program for Immunization review platforms across district stakeholders through digitalized data.

**Conclusions:**

Embedding digital technology into mainstream health systems relies on use by both end users and district stakeholders. Ease of operability, satisfaction with data reliability, personal recognition, links to field support, and empowerment are powerful enablers, whereas improved coordination as a result of easy, transparent data access can be an important by-product of digitalization. Findings are relevant not only for wide-scale implementation of immunization tracking apps in Pakistan but also for informing the use of digital technology for results-based delivery by frontline health workers.

## Introduction

There has been a recent spate of mobile phone–led programs, known as mobile health (mHealth) [1], to tackle health care issues such as immunization, tuberculosis (TB), and malaria in low- and middle-income countries (LMICs). Mobile phones are used by 97 per 1000 people in LMICs [2], reaching remote communities that previously had little interaction with public authorities or private companies [3]. In rural Tanzania, mobile technology has been used to provide the current inventory of anti-malarial medicine to improve access to drugs and other medical supplies [4]. In rural Uganda, an app called TB Detect enables health care providers to access TB-related educational material [5]. Another area where mHealth is being increasingly applied is routine immunization services in LMICs. SMS reminders about immunization and the use of electronic registries to trace cases who missed vaccination are being trialed in an increasing number of LMICs in Latin America and Sub-Saharan Africa [6-16]. There is some evidence that these programs improve vaccination completion rates [6,8-16]. However, many have not progressed beyond the pilot phase, and evidence for the effectiveness of digital solutions is limited [17].

Despite the proliferation of mHealth apps, not all digital health programs perform as intended, and assessments of the technology, health systems, and behavioral factors need careful consideration [18]. In particular, mHealth programs that rely on the performance of health workers would benefit from qualitative research to understand the extent to which end users and decision makers are prepared to engage with mHealth technology [19,20]. Experience from high-income settings emphasizes a user-centric approach to the development of digital technology [21]. This is also an important consideration to assess the implementation of these technologies within LMICs. Technological factors such as internet access, data volume, and app usability can affect implementation [22]. In addition, other factors such as personal motivations for data use, work culture, and health system support should be reviewed when adopting mHealth programs.

In Pakistan, 66% of children are completely immunized; this is much lower than the country target of 90% set by the Global Vaccine Action Plan [23]. Routine childhood vaccinations are provided by a dedicated cadre of government-employed vaccinators at static health centers and during outreach visits to villages >5 km from the health center. Poor vaccinator performance, which is exacerbated by poor district supervision, has been a chronic issue for Pakistan’s vaccine delivery system. A minimum of 1 vaccinator is allocated to each union council (UC), which is the smallest district administrative tier with a population of 10,000-20,000 people. Vaccinators are responsible for registering eligible children, providing vaccinations, updating Expanded Program on Immunization (EPI) cards, maintaining EPI records, and counseling parents for routine immunization awareness. The vaccinators are supplied with a motorbike, fuel support, and vaccines for routine immunization by the EPI program. UC–based EPI activities are administratively supervised by the district health office. However, paper records are poorly maintained and have questionable data. In addition, evidence suggests that vaccinators make too few outreach visits, provide little routine immunization education for parents, and maintain poor records [19]. Anecdotal evidence also suggests that vaccinators are resistant to supervision and often enjoy political patronage from local legislators protecting them from accountability on work performance [24]. In 2014, an app to track vaccinator movements through GPS was introduced across the populous Punjab province to ensure vaccinators made a sufficient number of outreach visits. Called e-Vaccs, it was designed, championed, and implemented by the government with provincially driven vertical accountability. Since then, policy commitment for digital immunization monitoring has also increased in other provinces, with the aim of effectively mobilizing the vaccinator workforce and moving away from relying on questionable paper-based records. However, a lack of the required systems support has stalled the roll-out process [20].

In this paper, we report the end-user experiences with a mobile app (Teeko, Aga Khan University Pediatrics Department, Pakistan) to track the delivery of routine childhood immunizations as well as the enablers and barriers for implementation in local health systems. Teeko is a mobile app that tracks vaccinators’ routine immunization performance. It was co-designed with the sub-national government as part of a larger health system strengthening research program for routine childhood immunization and piloted in a rural district of Sindh, Pakistan in 2015-2017. The app generates quality real-time data, and its key features include GPS tracking of vaccinator outreach visits, digital records of the immunization volume at the static health center and during outreach visits, creation of the next scheduled immunization encounter, and identification of children who missed vaccination. We drew on the experiences of Teeko usage by vaccinators and district managers, including acceptability of the app, use of the digitalized data, perceptions of the data validity, and enabling factors and barriers for its adoption. We aim to inform how digital technology can be embedded within district health systems in LMICS.

## Methods

### Overview

Our study explored the experiences with an Android-based immunization app to improve vaccination coverage in a rural, disadvantaged district of Pakistan. The overall objective was to evaluate the experiences of vaccinators and district managers during their use of the app to track routine immunization encounters. The specific objectives were to determine the acceptance and operability of the app as a tool for tracking vaccine encounters within the district health system, assess the data validity and data-related concerns of stakeholders, and understand how the technology is being used within the vaccine delivery system.

### Setting

**Figure 1:**
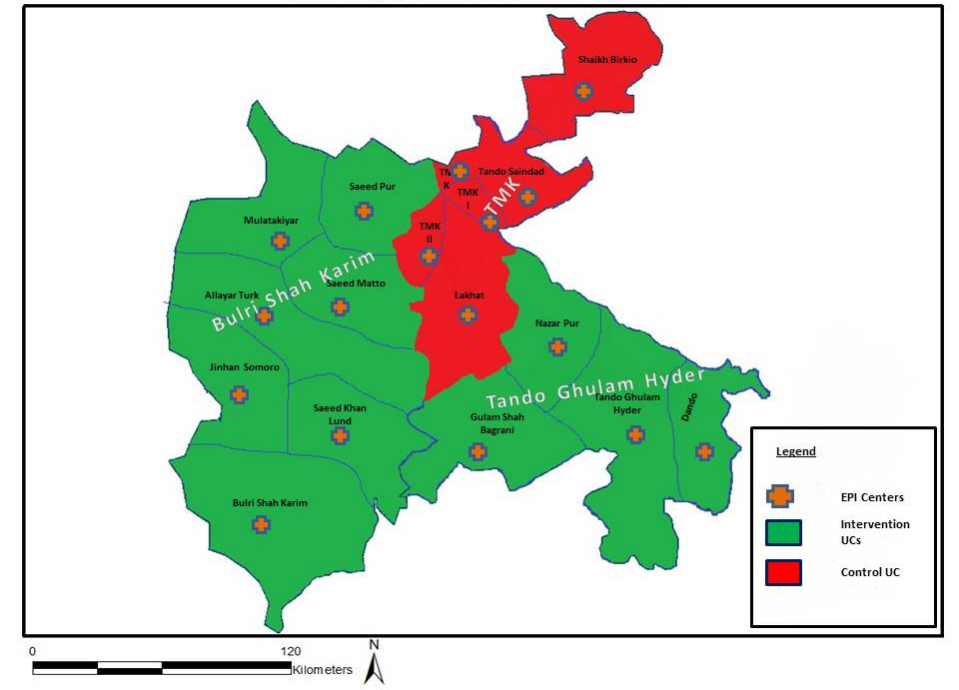
Map of the Tando Muhammad Khan (TMK) district in Sindh province, Pakistan, including locations of union councils (UCs) and Expanded Program on Immunization (EPI) centers.

The mHealth initiative was part of a larger implementation research program to strengthen the health system and routine childhood immunization conducted by the Aga Khan University (AKU) in collaboration with the provincial EPI in the Tando Muhammad Khan (TMK) district in Sindh Province in 2015-2017 (Figure 1). This district had poor vaccination performance, with a Penta coverage rate of only 23% and PCV coverage of only 11% at the start of the study. A package of integrated interventions including a digital app was implemented based on formative research to identify interventions that was conducted at the start of the study. In addition to the mHealth intervention, other complementary interventions included facilitation of a district EPI review platform, microplanning training, fuel support routed through the district health office for outreach activities, and co-financing of EPI motorbikes for UCs lacking functional bikes for outreach sessions. Interventions were implemented in 2 district sub-divisions (Talukas) comprised of 11 UCs. The Teeko intervention was implemented over 24 months, after which vaccinator and district stakeholder interviews were conducted. The immunization program resulted in a significantly higher number of completed vaccinations at the intervention sites than at the control sites, as measured in terms of PENTA3 and PCV3 coverage (Table 1).

**Table 1:** Improvement in the vaccine coverage rate from 2014 to 2017 during the Health Systems Strengthening (HSS) immunization pilot study [19].

Vaccine		Intervention, %	Control, %	Difference, %	*P* value
**PCV3**					
	2014	11	19	20	.001
	2017	44	32	—	—
**PENTA3**					
	2014	23	31	20	.001
	2017	44	32	—	—

### mHealth Intervention

**Figure 2:**
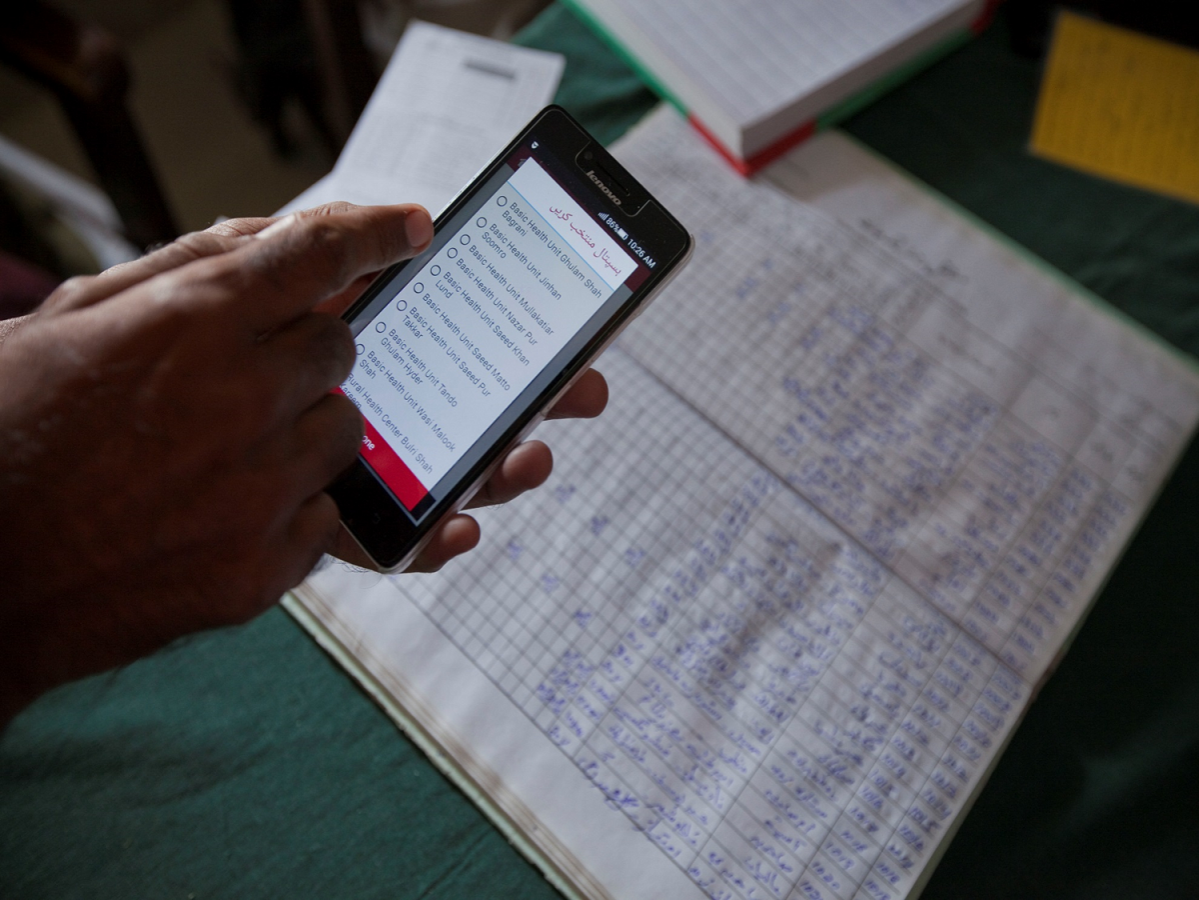
Vaccinator using the Teeko app.

Vaccinators and their sub-district and district EPI supervisors were provided with smartphones to track the immunization encounters. The app contained a list of UCs, villages, and health facilities assigned to each vaccinator. Vaccinators could access the app via unique access numbers to enter immunization data for the villages listed in their UCs (Figure 2). The app tracked vaccinator visits, vaccination volume at the static health center and outreach points, identified children that had missed a vaccination, tracked vaccine availability, and provided communication features for parents. The app had two components: data entry and a Web portal for data visualization.

The immunization app was developed over a 6-month field testing period in 2014-2015 and was implemented in 2015-2017. The principal investigator (SZ) conducted a baseline assessment of routine immunization delivery in the district and identified the reporting of incomplete, questionable immunization EPI data and poor use in the TMK district [19]. During the assessment, management information system records were reviewed, and EPI officials, vaccinators, health workers, and health facility managers were interviewed. Further follow-up meetings were held with EPI provincial staff, the district health team, and vaccinators by the principal investigator (SZ) and a research specialist (SH) to identify intervention points within the EPI record flow that could be supported with an immunization tracking app, the support required for vaccinators and district health officers to use the app, and the health system interventions required to facilitate decision making. An in-house technology firm (Aga Khan Development e-Resource Centre) was contracted for digital programming. App design and development were overseen by a steering committee chaired by the Provincial Director General of Health and comprised of the study principal investigator (SZ), a research specialist (SH), UNICEF, WHO, the EPI focal person for the TMK district, the District Health Officer for TMK, and a representative from the technology firm (SS). Using a participatory process, the following app features were identified: compulsory photo identification; defaulter (children who missed a vaccination) status using traffic light colors; alignment with EPI’s management information system; client data to include the parents’ national ID card numbers, household number, and village details; and adjustment for community migration. Meetings were held during the design phase and continued into the app roll-out, with recorded minutes. Iterations included the addition of new vaccines, identification of areas of misreporting, adjustment for vaccinations received outside the study area, and adjustment for household migration. To improve the technological features, the study team and technology partner field-tested different versions of the app with vaccinators and the district EPI focal person. Due to poor internet connectivity, the ability to upload data offline was added after the field testing. SMS messaging was poorly received and replaced with a robotic call. The study team randomly checked the photo entries and uploaded data for errors.

Finally, the app features included registration, immunization encounter record, offline data mode, vaccine stock management, awareness content, text messaging, a central database, and a Web portal.

#### Registration

The vaccinator registered the child at the first encounter, entering the child’s father’s name, child’s date of birth, household phone number, village name, and UC (Figure 2). This created an individual profile for the child. The child registration form was the same as the manually completed EPI child registration form. The data for the registered child was synced with the central database, in which all data were stored.

#### Immunization Encounter Record

**Figure 3:**
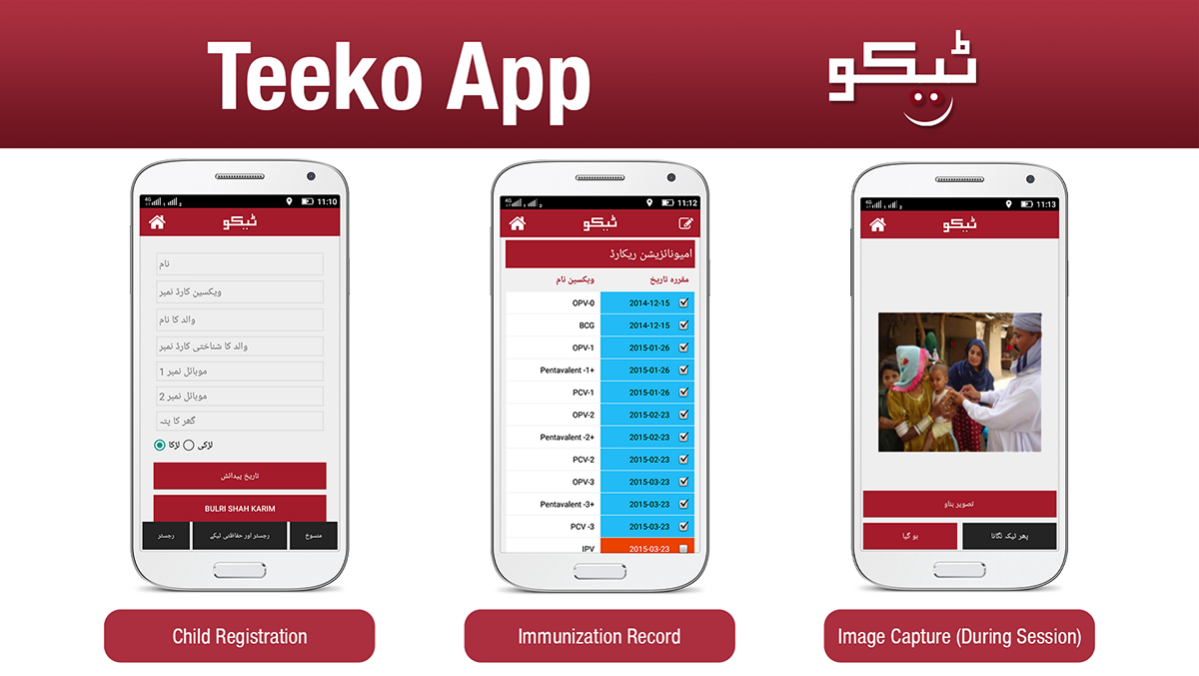
Examples of registration, an immunization record, and the image capture view on the Teeko app.

The vaccinator then vaccinated the child and uploaded a mandatory photo of the vaccination or updated EPI card (Figure 3) as proof of vaccination. A GPS-based location was generated for the vaccination site. The app sent the immunization record data to the central server in real time.

#### Offline Data Mode

The app’s offline feature allowed vaccination data to be recorded when internet connectivity was not available. The records were stored locally in the app and uploaded once an internet connection was restored during the outreach visit or at the health facility. Vaccinations could occur within the health facility or during an outreach visit.

#### Vaccine Inventory Management

To track inventory and ensure timely pick up of required supply, vaccinators could enter the amount of vaccine they had with them as well as the inventory at their respective health facilities.

#### Awareness Content

The app had certain communication features. A 60-second awareness video on routine immunization could be played by the vaccinator to educate the parents during a vaccination encounter.

#### Text Messaging

Following the vaccination, robotic calls and SMS messages about the routine immunization schedule and next visit were sent to the caregivers of each registered child.

#### Central Database

**Figure 4:**
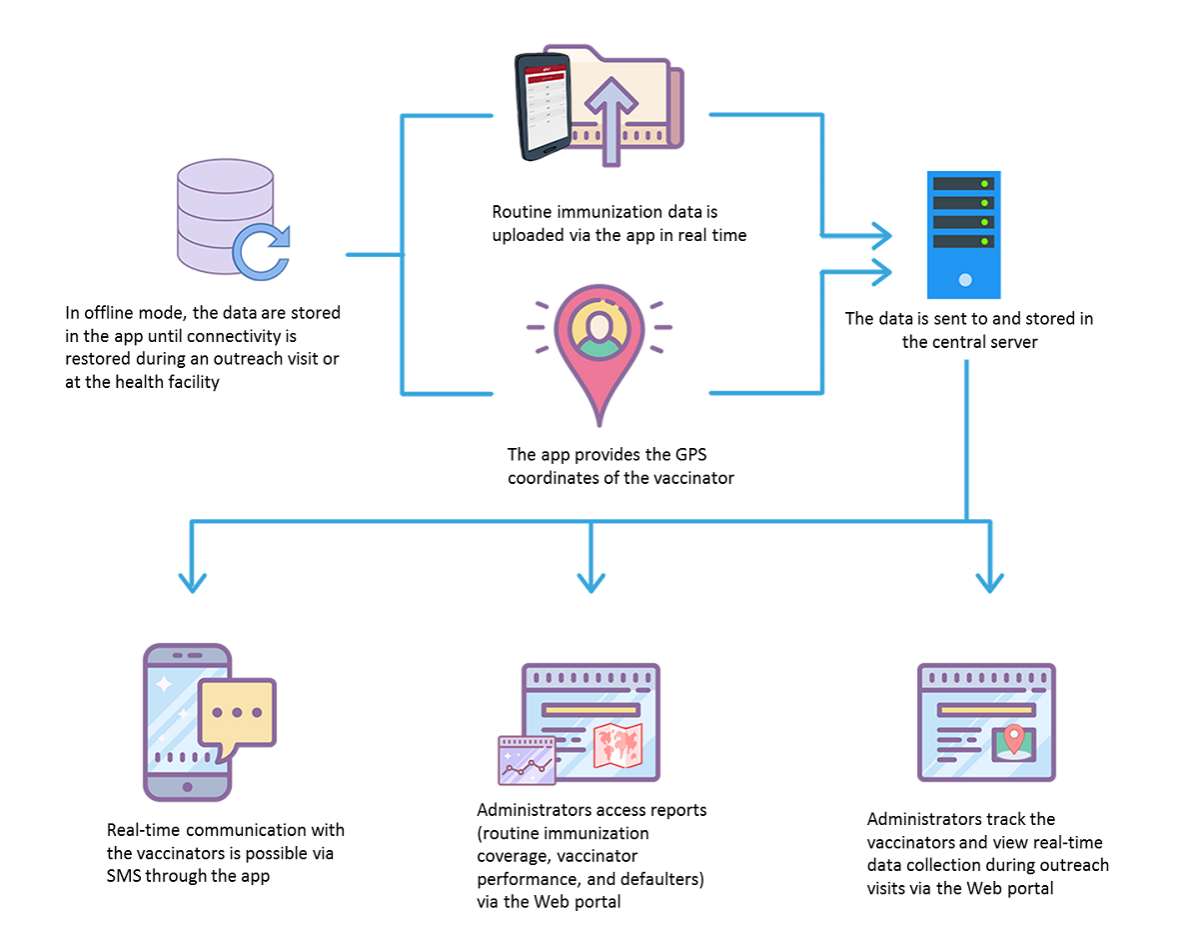
Flow of data through the Teeko app and Web portal.

**Figure 5:**
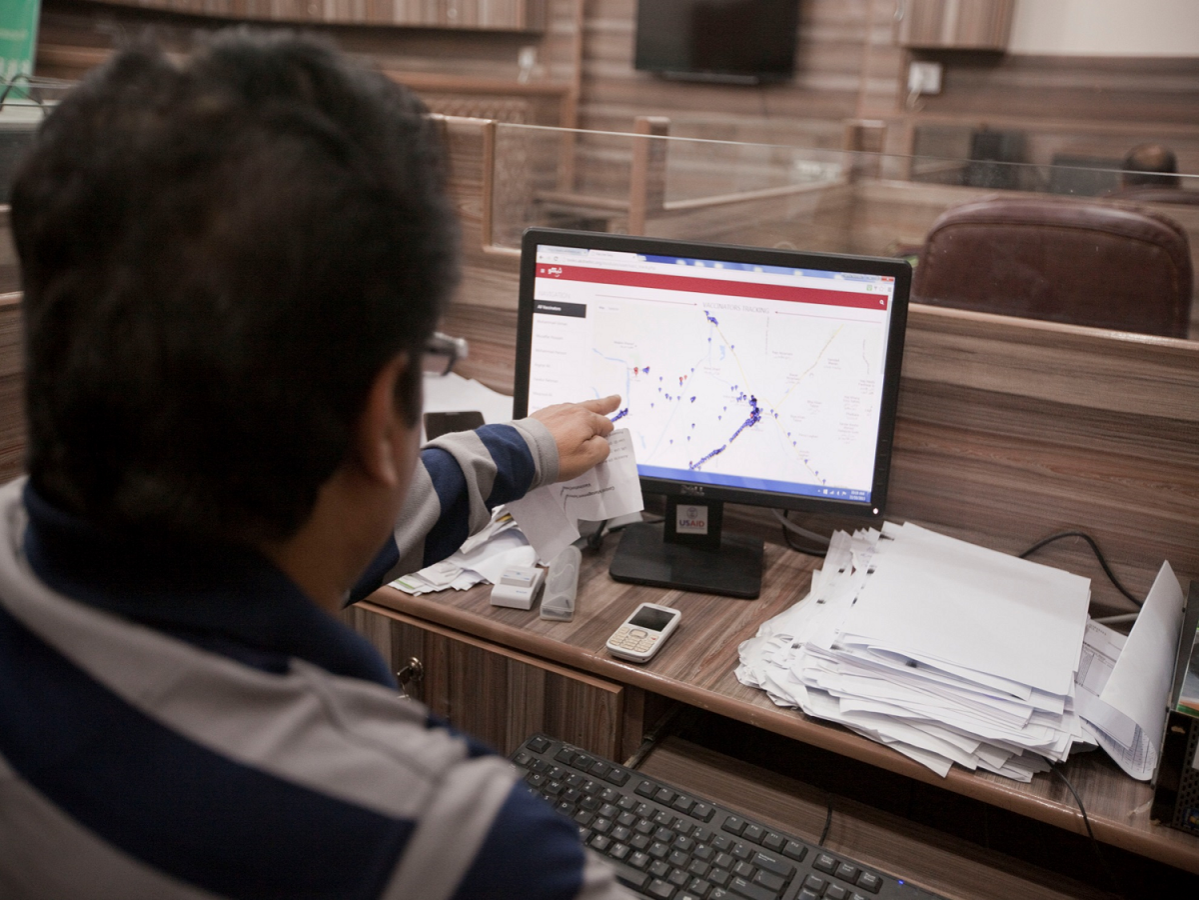
Vaccinator tracking screen in the Teeko Web portal.

The central system (Figure 4) allowed the administrator to register all users (vaccinators, lady health workers, and viewers). In addition to during the vaccination encounters, the app occasionally sent the GPS coordinates of the vaccinators to the central database (Figure 5). If internet connectivity was not available, the coordinates were stored in the local database and later uploaded to the central database. The vaccinator directory listed the UC assigned to each vaccinator.

#### Web Portal

Through the Web portal, the management and supervisory staff remotely monitored vaccinators’ activities and movements in real time (Figure 5). The SMS Panel allowed the administrator to send an SMS to the vaccinators and lady health workers about meetings, schedules, and data corrections. The portal also generated district and UC reports on routine immunization coverage, defaulters, and individual vaccinator performance. These reports were generated based on formats already in use by the EPI Program.

### Data Collection

We conducted semi-structured interviews with vaccinators and key informants (district managers) involved with EPI delivery (See Table 2). Data were collected and analyzed by trained researchers.

**Table 2:** Themes and tools used to explore the user experiences with digitalized immunization tracking and the key enabling factors

Themes	Tools	
	In-depth interviews with vaccinators	Key-informant interviews with district stakeholders
Acceptability and operability of the app	Use of key app features and app operability	Acceptability of the app within the district health system, enabling factors, and constraining factors
Validity of the app data	Perceptions of and concerns with the data validity	Perceptions of and concerns with the data validity
Use of the app data	Engaging with the app data, enabling factors, and constraining factors	Engaging with the app data, enabling factors, and constraining factors

#### Vaccinator Interviews

Semi-structured interviews were conducted covering the following topics: frequency of use of the app features; operability of and issues using the digital app; perceptions of the data validity and comparison with that of manually collected data; engagement with the app; and enabling factors and barriers for data use. Interviews were conducted with all 26 vaccinators working in the 11 UCs in which the digital app was implemented. All the vaccinators used the immunization app for at least 24 months. Individual interviews were conducted with each vaccinator in the local language and the privacy of a separate room.

#### District Stakeholder Interviews

In-depth interviews with key informants (district stakeholders) included the following topics: acceptability of the app to track immunizations conducted by the district heath team, perception of the data validity, and use of the information by district health management. For each topic, we also asked about how their work had changed after using the app and the enabling factors and barriers for app use. The topic guide was written in English and translated into the local Sindhi language. Interviews lasted 45 to 60 minutes and were conducted in the local language. Stakeholders from the district administration and district health office were purposively selected based on their key roles in EPI oversight or delivery. A total of 7 district stakeholders were interviewed, including the Deputy Commissioner (administrative head of the district and chair of the Polio-EPI district committee), Assistant Deputy Commissioner, District Health Officer, Assistant District Health Officer, District EPI Focal Person, District Superintendent of Vaccination, and Coordinator of the Lady Health Worker Program (rural community-based female health workers that assist with the awareness provision for routine immunization and mobilization of households for routine immunization sessions provided by vaccinators).

### Ethical Considerations

Ethical approval for the study was obtained from the Ethical Review Committee of Aga Khan University Karachi, Pakistan (ERC number: 2818-Ped-ERC-13). Informed consent was obtained from all participants. Interviews were conducted on a voluntary basis. Each participant was ensured of the confidentiality of personal information. Personal information was anonymized for analyses and reporting through a coded interviewee number. The recorded data were kept in locked drawers and encrypted laptops to ensure data confidentiality.

### Data Analysis

The interview information was transcribed in Sindhi and translated back into English. These were then checked for errors and manually analyzed. Three a priori themes were identified in line with the research objectives and used as the basis for inductive analysis: acceptability and operability of the app, data validity, and use of the app data (Table 2). Content analysis of the transcripts was undertaken, developing main codes in line with the a priori themes and further detailed coding based on grounded findings emerging from the narrative.

## Results

Interview guides were used to report the results from the in-depth interviews. Textboxes 1 and 2 highlight the important findings from the vaccinator and district management interviews, respectively.

### Vaccinators’ End-User Experience

Table 3 lists specific responses provided by vaccinators during their interviews.

#### Acceptability of the App

#####  Use of App Features

All 26 vaccinators used the app to record their vaccination encounters. Nearly all vaccinators (n=24) took post-vaccination photos of the children. Although some parents initially refused the photos, they eventually agreed after the vaccinators explained that the photo would be used for immunization verification and recording. Only two vaccinators reported that parents would not allow a photo under any circumstances, in which case a photo of the vaccination card was uploaded instead. These vaccinators were assigned to UCs that had pockets of the population with reports of prior vaccine refusal.

All the app features for routine immunization awareness including the awareness video, SMS alerts, and robotic calls were used by 18 vaccinators. Only the awareness videos were used by 6 vaccinators, and 2 vaccinators used only the SMS alert and robotic call features.

##### Operability

Of the 26 vaccinators, 25 reported that the app was easy to use and they could operate it without difficulty (Table 3). One vaccinator, who had a stroke-induced disability, needed his son’s help to enter the data in the app. However, he also required assistance to manually complete data entry. Real-time data display in the format of the government EPI report was considered helpful for reading and relating to the data.

Vaccinators reported no instances of data loss from the app. Internet connectivity issues often occurred in the more remote villages, but the app allowed offline data entry. Then, vaccinators uploaded the data after they returned to the facility. The offline feature was particularly appreciated for its ability to prevent data loss (see Figure 4).

However, a few vaccinators reported being uncomfortable using the offline option and would have preferred real-time information uploads to the central database. Their reported concern was they might forget to upload pending data or be unable to upload pending data if the phone was damaged or stolen. However, there were no reports of phone damage or loss during the study.

Another reported issue was that, as the phones aged over the 24-month period, the phones would hang or freeze while using the app, making it difficult to enter data. Often, the phone had to be restarted, and there was a concern that unsaved data would be lost. The vaccinators wanted a replacement phone at least every 2 years to continue using the app.

#### Validity of the App Data

Most of the vaccinators (n=23) reported a high likelihood of error with manual documentation of outreach vaccination encounters. In practice, the data were entered based on recall in the EPI registers at the health care facilities rather than on site. Vaccinators recounted poorly recorded field data during prior measles outbreaks (before the Teeko app was available), resulting in an inability to target defaulters. They perceived that instant data entry through a mobile app generated a more accurate defaulter list.

**Important findings from the vaccinator interviews.**

**Acceptability of the App: Use of App Features**

- All vaccinators used the app for vaccination encounters.
- Photos of the immunized child were uploaded more frequently than photos of the vaccination card.
- Most vaccinators used the videos, SMS, and robotic calls for immunization awareness education and routine follow-ups.
- Some vaccinators used only the videos for immunization awareness education.

**Acceptability of the App: Operability**

- Vaccinators considered it easy to operate.
- They could upload the data in villages without waiting for internet connectivity.
- The vaccinators preferred using the app over manual data entry.
- Issues included hanging of the phones after 2 years.

**Validity of the App Data: Perceptions of Data Validity**

- The vaccinators believed the accuracy of child registration and vaccination status was better than that of manual records.
- The photo verification was considered a key feature for accuracy.
- The vaccinators felt as if they were more likely to follow the monthly plan for visits because their supervisors monitored them via GPS tracking.

**Validity of the App Data: Engagement With the Data**

- Vaccinators could proactively identify and track defaulters.
- They could develop a monthly visit plan that could particularly target defaulters.
- Their performance could be tracked accurately by their supervisors using GPS tracking.

**Use of the App Data: Enabling Factors**

- Reporting of individual performance resulted in supervisory recognition and personal gratification in reaching targets.

**Use of the App Data: Barriers**

- Data entry via both the app and manual methods resulted in double the amount of work.

**Important findings from the key informant interviews with district management.**

**Acceptability of the App: Acceptability Within the District Health System**

- The vaccinators were comfortable using the app.
- The Expanded Program for Immunization (EPI) supervisors found it easy to retrieve the data to monitor vaccinators and identify defaulters.
- The key informants requested to continue using the app and expand its use to other districts.
- The district was able to act as an example for other districts.

**Acceptability of the App: Enabling Factors**

- The district managers were interested in the app and expressed ownership of it.
- Data-driven accountability led to coordination across health programs and with the district administration.
- Data-driven accountability resulted in a demand for work recognition as well as fear of exposure.

**Acceptability of the App: Barriers**

- Additional effort to increase awareness of routine immunization was needed in high-defaulter areas.

**Validity of the App Data: Perceptions of Data Validity**

- GPS location of vaccinators provided reliable information, while previously checks were onerous.
- Vaccination volume and child details were very accurate and could be quickly verified.

**Validity of the App Data: Engagement With the Data**

- Real-time data was used to inform EPI monthly review meetings about the increasing vaccination volume and reduced number of defaulters.
- The monthly recognition of high-performing vaccinators and naming the poorly performing vaccinators encouraged sharing of experiences.

**Use of the App Data: Enabling Factors**

- The data enabled coordination across the district stakeholders.
- The individualized public recognition was considered the biggest motivator.
- The data encouraged competition among the vaccinators as well as sharing of lessons learned.
- The app data were used to release mobility support.

**Use of the App Data: Barriers**

- Political patronage is still an issue but better managed due to transparency of accountability and monitoring of the vaccinators in the target setting.

**Table 3:** Specific responses from the vaccinator interviews, organized by theme.

Theme	Examples of vaccinator responses
Acceptability of the app: Operability	“It is very easy to use, just like my own phone that I use, typing information on the phone is easy too... and we already knew how to take a picture and upload it...”
	“I never lose any of the data now, as the data is entered then and there in the outreach, ...first we used to write it on a piece of paper and bring it home and then enter it the next day at the health facility...there was a huge chance of error... “Teeko” data is very reliable data”
	“The application can save my data until I get internet signals but still, to avoid error due to this in future, a better connectivity in remote areas is needed...”
	“It’s been two years now, mobiles phones should be replaced with new ones, ... they hang and shut down at times... Taking more time to make an entry in the field or transfer the data...”
Validity of the app data	“It is easy for me to find children who are overdue for their vaccination, I can see the due date for the next vaccination and reach the child”
Use of the app data	“The district administration has become more watchful and alert now... they can catch us not being in the field through their mobiles. now I believe that I am performing my duty with more honestly than I used to previously...”
	“During the last award ceremony, I was awarded first position by district-AKU team; my data recorded on “Teeko” application in the field also completely matched the manual data...I was overwhelmed”
	“I have been working for the last 35 years as a vaccinator and I feel that these two years were the ones where I performed my best. I feel very proud of myself”
	“Very easy application, but with manual (entry of records), it doubles my work; we have to make entries in both EPI (paper) registers and “Teeko”; this takes a lot of time”

The post-vaccination photograph was considered key to ensuring reliable data. Compared with the prior practice of checking the EPI records, the advantage of the photo feature was child verification. Vaccinators also reported that the GPS tracking of outreach visits resulted in greater vaccinator vigilance in conducting outreach sessions and improved the reliability of vaccination encounter reports. Vaccinators reported calls from their supervisors to check their location when the supervisors did not observe any field activity.

#### Use of the App Data

All vaccinators reported they mainly used the digital data to identify children overdue for their vaccinations. Vaccinators expressed ownership of the data and a feeling of empowerment when using the data to plan their monthly vaccination rounds. They also reported using the data to set target vaccination volumes.

All vaccinators also mentioned the use of the GPS feature by the district health office and deputy commissioner for vaccinator supervision. Vaccinators reported being questioned by the district supervisors if they turned off their mobile location or could not be seen moving in the field.

The vaccinators reported that the overriding motivator to use the app was the reporting of the performance level of each vaccinator. The vaccinators reported feeling gratified when the best performing vaccinators were publicly recognized during the review of individual performance levels in the monthly EPI meetings. All the vaccinators found it very satisfying to be noticed and praised by superior EPI officials and district officers. Most of the vaccinators reported that this motivated them to do their best. Others reported this was the first time they took their work seriously and found it a novel but satisfying experience to work toward targets rather than working to identify loopholes to avoid work.

A few vaccinators reported that the app increased their workload because they were also expected to manually document the EPI records in addition to documenting via the app.

Migration to digital records from manual recording was preferred by 23 of the 26 vaccinators, while 3 vaccinators wanted both manual and digital record keeping. All 26 vaccinators were willing to continue using the app in the future.

The vaccinators also mentioned the conditional fuel support provided by the district health office based on the number of monthly visits reported by the app. The vaccinators reported that the lack of fuel support made it difficult to conduct outreach sessions.

### District Stakeholder Experiences

Findings from the key informant interviews with district stakeholders were categorized into the following thematic areas: acceptability of the app for routine immunization supervision by the district heath team, perception of data validity, and use of the app information by the district health management. Within each thematic area, the narrative reports were analyzed for changes in work after using the app, experiences with implementing the app, and the enabling factors for and barriers to app use. Table 4 lists specific responses provided by the district stakeholders during their interviews.

**Table 4:** Specific responses from the district stakeholder interviews, organized by theme.

Theme	Examples of district stakeholder responses
Acceptability of the app	“Good coordination between the Health and District Administration is seen, District Health Office helped AKU to pilot and develop “Teeko” while the Deputy Commissioner Office supported in making their planning work, also did field monitoring and addressing administrative issues”
	“Commissioner (district) requested to start the same project in his district, we were called to give a briefing on “Teeko” he was very impressed...”
	“Under the Health Department, EPI Provincial Program should adopt “Teeko” immediately...at least in the districts which are not performing up to mark”
Validity of the app data	“At first we used to follow vaccinators schedule, get hold of someone who knew the area, then try to find him in the field, now we see his location in “Teeko” application and reach him...it really saves a lot of time”
	“On looking at the data I send direction to vaccinators through “Teeko” (ap-plication) to correct it...they reply after getting it done”
Use of the app data	“If we took any disciplinary action against irregular or poor performing vaccinators, we faced political pressure at times and are threatened too, we then become helpless...”
	“Political involvement has always been an issue, but with sincere working, like with this “Teeko” project, we can overcome it.”
	“At the office of Deputy Commissioner, in a formal ceremony with heads from the Teeko project and District Health Officer the best performing vaccinators were appreciated, they were given certificates, shields and gifts...everyone wanted to do more to earn this honor and respect...some of the vaccinators were so motivated that they even worked on Sundays to meet their target”
	“The vaccinator’s data is assessed in the EPI meeting for future decisions & micro planning.”
	“The vaccinators were offered motorcycles, they had to pay 50% while the project paid another 50%, it not only solved mobility issue, they owned a motorcycle as well...every month they (Vaccinators) get POL from District Health Office...the accountability made possible regular POL supply to vaccinators, which previously was not provided.”
	“With mobility support and real monitoring, the routine immunization has improved in the outreach”

#### Acceptability of the App

Key informants from district management commonly reported that the vaccinators were comfortable using the app. District officials found it easy to retrieve routine immunization volume from their cell phones and computers, and the officials used the app to keep track of children who missed immunizations. The interviewees recounted poor vaccinator performance prior to using the app and attributed this to poor work attitudes, insufficient supervision from the supervisors, and a lack of fuel support and functional motorbikes for supervisory visits.

The stakeholders reported that the app led to increased coordination between the EPI and Lady Health Worker Program—the two important community outreach resources within the district heath system—and closer interaction regarding routine immunization between the District Health Office and the Deputy Commissioner’s Office.

The health officials expressed an eagerness to continue the tracking initiative and anxiety that routine immunization coverage would decline again after closure of the project. The stakeholders also requested that the project be expanded to the other sub-divisions within the district. The district leadership reported feeling as if they could serve as an example for other districts in the province and were gratified that other districts requested demonstrations and sharing of lessons learned.

#### Validity of the App Data

Key informants reported that tracking a vaccinator in the field was challenging in routine practice. Immunization work plans provided by the vaccinators were not always accurate; they could make changes to the plans without prior notice to the district health office. Moreover, even when EPI supervisors verified the vaccinators’ locations in the field, they needed assistance from local villagers familiar with the area. It was still possible that they would not successfully find the vaccinator. The district stakeholders appreciated that the app facilitated vaccinator tracking through its GPS feature. They were able to locate and reach vaccinators without delay.

The EPI district supervisors reported that they relied on the app data to provide supervisory instructions for the vaccinators related to routine immunization coverage and defaulter reports. The app data were considered superior in quality to manually collected data and were particularly used for corrective actions.

#### Use of the App Data

The key informants commonly reported that the main uses of the app data were improving the number of routine immunization encounters and locating defaulting children. In the past, it was challenging to hold the vaccinator cadre accountable for routine immunization performance, making it difficult to improve vaccination volume. Furthermore, disciplinary actions such as a “notice of explanation,” for which employees must provide an explanation for an allegation of violation of company policies, rules, and procedures; “show cause letter,” which asks the employee to provide a reason why they should not face a disciplinary action for a conduct or capacity issue in the workplace; and termination of irregular, ghosting, or nonperforming vaccinators were not effective due to the political patronage of vaccinators by local legislators.

The key informants mentioned that regular EPI review meetings were convened at the district health office, and the meetings used the real-time app data. These meetings were often attended by the district health commissioner and assistant commissioner. During these meetings, vaccinator performance was reviewed, including that of poor performers. Vaccinators with high performance were also recognized separately in quarterly public ceremonies attended by district legislators. The stakeholders reported that the public recognition was instrumental for increasing motivation. They added that the review meetings provided a platform for vaccinators to learn from each other’s experiences and promote healthy competition.

District stakeholders also expressed a sense of empowerment from using the app to make operational decisions to improve routine immunization in the district. They used the data to direct activities to areas with incomplete vaccination coverage. Real-time immunization data were also used to provide support for vaccinator mobility based on GPS-verified outreach visits.

Additionally, EPI motorbikes were provided by the district health office to the better performing vaccinators; these were co-financed by the project and the vaccinator. Both activities were undertaken during the monthly district EPI review meetings that were chaired by the District Health Officer.

## Discussion

In LMICS, mHealth apps are increasingly used for immunizations and other public health concerns; however, recent evidence has largely focused on app development, dashboards, or the before-and-after effects on awareness or coverage [6,7,10,12,21-25]. Less evidence is available regarding end users’ and decision makers’ acceptance of and engagement with digital health technology. Bridging this evidence gap is critical to embed digital technology into mainstream health systems. In this paper, we report the qualitative experiences of frontline health staff and district managers in engaging with real-time, digital technology to improve the coverage of routine childhood immunization in an underserved rural district in Pakistan.

Using an iterative approach, we gathered information about the acceptability of the app for data collection and tracking vaccine delivery, perceptions about data validity, the extent that app data were used, and the engagement of care. We also identified the key factors for frontline staff to engage with digital technology. The app was commonly accepted by vaccinators and district supervisors to track vaccination encounters; however, the app features aimed at increasing parents’ vaccination awareness were less commonly used. Lack of parental agreement for photograph verification of their children was not a major issue. Vaccinators found the app easy to operate, the fields were similar to those of EPI records, and the offline data recording feature was helpful in remote access areas. Double entry of both manual and digital records was considered time consuming, and aging phone technology created systems issues. District supervisors found the app data easy to access for quick verification of immunization activity and to provide supervisory direction. The ability for real-time tracking of both individual vaccinators and the entire team through the Web portal considerably reduced the time spent tracking vaccinators during outreach visits. This helped to not only accurately track the vaccinator but also identify vaccination coverage according to the geographical location. This drastically reduced the monitoring and tracking time during outreach. The increased availability of supervision time was then utilized for other monitoring and evaluation activities. In addition, the app-generated maps helped to finalize microplans and identify missed locations. The validity of the app data was considered superior to that of data from manual records by both the vaccinators and district supervisors; this perception was attributed to photo verification of the encounters. Opinions differed regarding what contributed to the improved data. The EPI supervisors attributed it to the transparency of vaccinator movements, while vaccinators felt that the ability to record immunization entries during the visits was better than using recall to manually record data later at the health facilities.

Real-time immunization data were mainly used to monitor vaccination volume, track children with incomplete vaccinations, develop outreach visit plans, alter existing microplans, and provide a results-based fuel allowance. A significant outcome was the initiation of regular monthly EPI review meetings by the district leadership. During these meetings, real-time data were used to review immunization progress. Data were also used to recognize both good and poor immunization performance per individual and across UCs and vaccinators. These findings are supported by those from a study in Nigeria that used GPS and geospatial data to track vaccinators [26,27]. They reported improvements in microplanning, monitoring, and immunization coverage, which were attributed to the technology-based support for performance monitoring that reduced erroneous entries and data fabrication by vaccinators. They also used GPS data to provide feedback to the outreach team and target missed geographical areas.

Our study also identified enablers that facilitated the use of digital immunization tracking. Recognition of individual performance, empowerment, and results linked to fuel support emerged as powerful motivators for the vaccinators to work towards target delivery rather than avoiding workload, as they had previously. For district management, key enablers included a sense of empowerment for reviewing and planning EPI delivery and enhanced transparency to counteract political patronage of vaccinators. A knock-on effect of the real-time verification data was improved coordination across different components of the district health systems.

Reviews of digital technology use in other settings have drawn on mixed methods including interviews, focus group discussions, and systems analysis of the app data. The evidence indicates that instant data availability for monitoring purposes, as observed with neglected tropical diseases [28], and ease of use and digital literacy of end users, as observed with oral cholera vaccinations [29], are important parameters for the successful trialing of digital apps. Poorly designed devices and inadequate cellular infrastructure have been noted as barriers for integrating apps into health systems [30]. Lessons learned from immunization registries in Latin American and Caribbean countries indicate that digitalization must be useful to vaccinators for the data to be of good quality. Evidence on non-technological factors for health provider apps is still emerging. A study of a client data app for community health nurses in Ghana resulted in reasonably good acceptability due to the app’s capacity to facilitate client follow-up and data reporting; however, the feasibility and usability of the app were hindered by high client volumes, staff shortages, and software and device challenges [31]. A study on an mHealth app for midwives in Ghana found low levels of user acceptance and a software design that did not match the end‐user needs or work environment [32]. Another study that introduced an app for community health volunteers in Kenya found that acceptability improved after a period of initial anxiety; however, there were feasibility challenges related to battery drainage and difficulty keeping the phone charged [33]. Yet another study in South Africa with an app for mHealth workers to electronically track patients with multi-drug resistant TB found a high intent for use but low actual usage resulting from forgetfulness and low levels of responsibility for such work [34].

Our study provides timely evidence on the successful integration of an immunization tracking app to be used by vaccinators as Pakistan prepares to upscale digitalized immunization tracking. It also provides lessons for LMICS on technological and non-technological factors that require attention to contextualize health provider apps within the local health system. We contend that immunization performance tracking through digital technology needs essential acceptance by frontline health force, and mere vertical enforcement is not enough. Technological ease of operability, personal recognition, results-oriented mobility support, and empowerment to improvise microplanning are powerful individual-level factors, whereas data transparency and empowerment for district planning are key organizational-level enablers to embed digital tracking in vaccine delivery systems. While there is a proliferation of digital technologies in countries such as Pakistan, dedicated investment in e-governance for continuous, independent, rigorous evaluation is required to assess actual use in the field and validity of the data.

### Strengths and Limitations

This study used iterative qualitative research methods for in-depth exploration of the digital technology interface with end users and decision makers within the health system. This iterative approach helped identify themes in an unexplored area and identified details that would have been missed in a more prescriptive quantitative study. Triangulation of the findings across vaccinators, supervisors, and district managers strengthened the analysis and helped draw out common convincing narratives. To limit bias, researchers who were not part of the intervention team undertook tool development and data collection.

A limitation of this study is the lack of quantitative assessment of the data generated by the app. A household vaccination coverage survey conducted as part of the larger health system strengthening immunization study found a significant difference in vaccination completion rates between the baseline and end timepoints across study control and intervention areas [35]. However, the app’s impact cannot be separately quantified from the other components of the larger health systems intervention package. While several of the findings are specific to the study context, the study provides the key parameters for consideration during the current rollout of digital immunization interventions in Pakistan as well as lessons for the introduction of a health provider app in LMICS. Digital technology investments must be accompanied by independent rigorous evaluation for relevance to health systems.

### Conclusions

Embedding digital technology into mainstream health systems relies on acceptance by both end users and district stakeholders. Ease of operability, satisfaction with reliable data, personal recognition, results-based field support, and empowerment are powerful enablers. An important by-product of digitalization can be improved coordination as a result of transparent, easy data access. The findings are relevant to not only the current upscaling of digital technology to track immunization in Pakistan but also the application of digital technology for results-based delivery by frontline health workers.

## Abbreviations

EPI: Expanded Program on Immunization
LMIC: low- and middle-income country
mHealth: mobile health
TB: tuberculosis
TMK: Tando Muhammad Khan
UC: union council
